# A small molecule inhibitor of ER-to-cytosol protein dislocation exhibits anti-dengue and anti-Zika virus activity

**DOI:** 10.1038/s41598-019-47532-7

**Published:** 2019-07-29

**Authors:** Jingjing Ruan, Hussin A. Rothan, Yongwang Zhong, Wenjing Yan, Mark J. Henderson, Feihu Chen, Shengyun Fang

**Affiliations:** 1 Anhui Medical University School of Pharmacy, Hefei, Anhui 230032 China; 2 Center for Biomedical Engineering and Technology, Department of Physiology, Department of Biochemistry and Molecular Biology, University of Maryland School of Medicine, Baltimore, MD 21201 United States; 3 National Center for Advancing Translational Sciences, National Institutes of Health, Rockville, MD 20850 United States; 4 Present Address: Department of Biology, College of Arts and Sciences, Georgia State University, Atlanta, GA 30303 United States

**Keywords:** Mechanisms of disease, Pharmaceutics

## Abstract

Infection with flaviviruses, such as dengue virus (DENV) and the recently re-emerging Zika virus (ZIKV), represents an increasing global risk. Targeting essential host elements required for flavivirus replication represents an attractive approach for the discovery of antiviral agents. Previous studies have identified several components of the Hrd1 ubiquitin ligase-mediated endoplasmic reticulum (ER)-associated degradation (ERAD) pathway, a cellular protein quality control process, as host factors crucial for DENV and ZIKV replication. Here, we report that CP26, a small molecule inhibitor of protein dislocation from the ER lumen to the cytosol, which is an essential step for ERAD, has broad-spectrum anti-flavivirus activity. CP26 targets the Hrd1 complex, inhibits ERAD, and induces ER stress. Ricin and cholera toxins are known to hijack the protein dislocation machinery to reach the cytosol, where they exert their cytotoxic effects. CP26 selectively inhibits the activity of cholera toxin but not that of ricin. CP26 exhibits a significant inhibitory activity against both DENV and ZIKV, providing substantial protection to the host cells against virus-induced cell death. This study identified a novel dislocation inhibitor, CP26, that shows potent anti-DENV and anti-ZIKV activity in cells. Furthermore, this study provides the first example of the targeting of host ER dislocation with small molecules to combat flavivirus infection.

## Introduction

Flaviviruses, such as dengue virus (DENV) and Zika virus (ZIKV), have expanded their geographic distribution due to changing climate, virus evolution, and social factors^1,2^. DENV is the most prevalent flavivirus and infects approximately 390 million people each year, of which 96 million manifest symptoms. Individuals who develop dengue haemorrhagic fever have a mortality rate of 2–5%^1,2^. ZIKV is also associated with severe neurological complications, such as microcephaly in newborns and Guillain-Barré syndrome in adults^3–5^.

Vaccination is the primary public health strategy to combat flavivirus infections; however, numerous challenges exist along the path from the development to the delivery of tolerable and effective vaccines^4^. An example of the effects of these challenges is the recent suspension of the DENV vaccine Dengvaxia in Philippines, which was developed by Sanofi Pasteur, after its brief clinical use because of potentially deadly side effects^5,6^. Significant outbreaks of yellow fever virus (YFV) in Africa and South America have been observed since 2015 despite a reliable vaccine for YFV^7^. Notably, the ZIKV epidemics mostly occurred in DENV-endemic areas, increasing the incidence of co-infection with these two flaviviruses. Thus, development of an effective vaccine is complicated because of the existence of different DENV serotypes and the possibility of pre-existence or co-existence of ZIKV infection, which creates an intricate immune response^8^. Alternate strategies, such as treatment with a broad-spectrum antiviral drug, could represent a valid approach to circumvent the problems associated with vaccines^9–11^.

Targeting essential host factors required for flavivirus replication represents an attractive approach for discovering antiviral drugs^9–11^. The Hrd1 ubiquitin ligase-mediated endoplasmic reticulum (ER)-associated degradation (ERAD) pathway may be one such host factor^12–15^. ERAD is a protein quality control mechanism in the ER that is mediated by ER membrane-anchored ubiquitin ligase complexes, such as the Hrd1 complex^16–20^. ER chaperones and lectins identify misfolded proteins and deliver them to ER membrane-anchored ubiquitin ligase complexes, following which misfolded proteins are transported across the ER membrane to the cytosol for ubiquitination and subsequent degradation by the proteasome^16^. Therefore, this ER-to-cytosol protein transport process, known as retrotranslocation or dislocation, is an essential step for ERAD^16,17^. Recently, genome-scale RNAi and CRISPR-Cas9 screens have led to identification of many crucial host factors for flavivirus replication, including several key regulators of the Hrd1-mediated ERAD pathway^12–15^. Based on this information, we hypothesized that small molecule inhibitors of Hrd1 complex-mediated dislocation would have broad-spectrum anti-flaviviral activity. To test this hypothesis, we screened small molecule libraries obtained from the NCI Developmental Therapeutics Program (https://dtp.cancer.gov/) and identified compound 26 (CP26), 1-methyl-4-[(2,3,4,5-tetrachlorocyclopenta-2,4-dien-1-ylidene) methyl] benzene (NSC26112, MLS000738051) as a dislocation inhibitor with potent anti-DENV and anti-ZIKV activity. This study provides the first example of the use of small molecules to target host ER dislocation to combat flavivirus infection.

## Results

### Identification of small molecule inhibitors of dislocation

**Figure 1:**
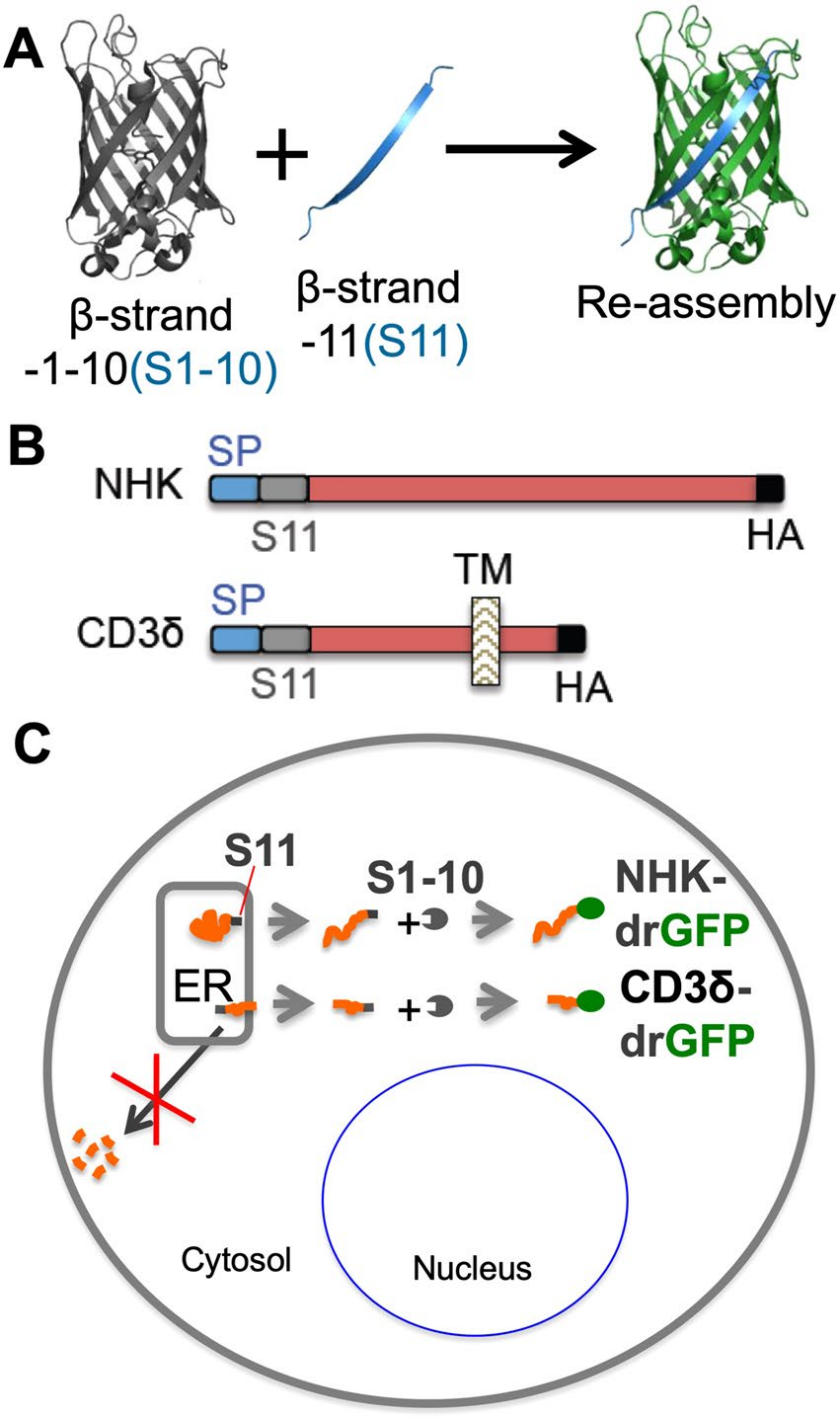
Detection of protein dislocation by dislocation-dependent reconstituted GFP (drGFP) reporter assay. (**A**) Split GFP technology. (**B**,**C**) Schematic representation of the split GFP-based method used to monitor ER protein dislocation. S11-tagged NHK or CD3δ is expressed in the ER, and S1–10 is expressed in the cytosol. GFP is re-assembled when S11-tagged proteins are dislocated to the cytosol and not degraded. Therefore, the assay is performed in the presence of a proteasome inhibitor to prevent the degradation of dislocated S11-tagged substrate. SP: signal peptide. TM: transmembrane domain. HA: haemagglutinin-derived peptide tag.

**Figure 2:**
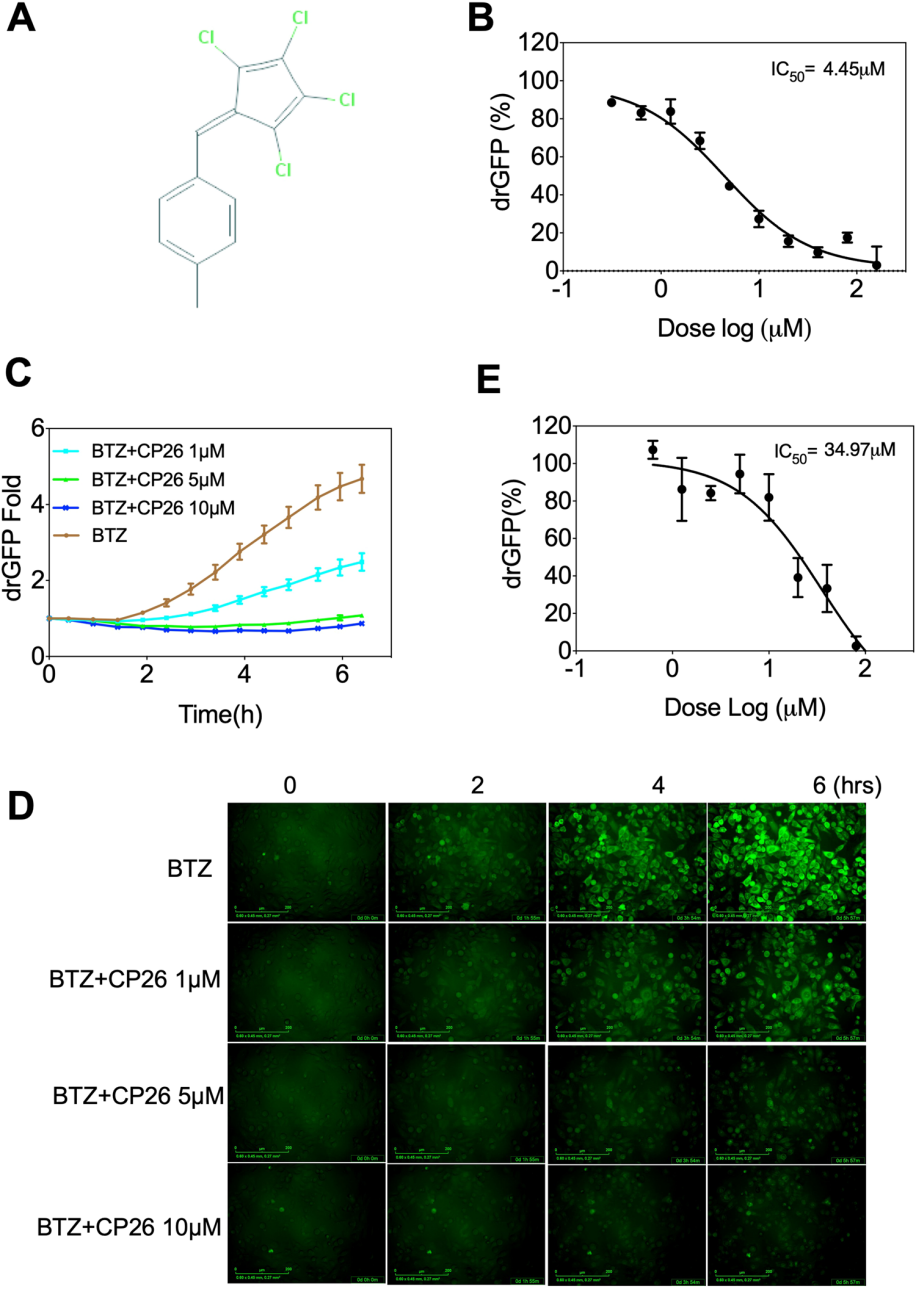
CP26 inhibits protein dislocation in the drGFP assay. (**A**) The chemical structure of CP26. (**B**) CP26 inhibited NHK dislocation in HeLa cells with an IC50 of 4.45 μM in the drGFP assay. (**C**,**D**) Time-lapse imaging of NHK dislocation in HeLa cells showing the dose-dependent inhibition of NHK-drGFP by CP26 (**C**: quantification of drGFP; D: drGFP fluorescence). (**E**) CP26 inhibited CD3δ dislocation with an IC50 of 34.97 μM in the drGFP assay. HeLa cells expressing NHK-drGFP or CD3δ-drGFP were treated as indicated, and drGFP intensities were measured in 96-well dishes and are expressed as the mean ± SEM, *n* = 3, (**B**,**C**,**E**) or imaged (**D**). BTZ: bortezomib.

We previously established a drGFP assay used to visualize and quantify ER protein dislocation in live cells^21^. drGFP was designed by taking advantage of previously reported split-GFP technology, in which GFP is separated into two fragments: one containing β-strands 1–10 (S1–10) and another containing β-strand 11 (S11). These two fragments can reconstitute GFP fluorescence when they meet^22,23^. To monitor dislocation of the mutant ER proteins, we established HeLa cell lines that stably express a mutant form of α-1-antitrypsin (NHK variant) or CD3δ tagged with S11 in the ER and S1–10 in the cytosol. When S11-NHK or S11-CD3δ are dislocated to the cytosol and their degradation is inhibited by a proteasome inhibitor, their S11 tag binds to S1–10 to reconstitute GFP fluorescence (Fig. 1). When dislocation is inhibited by a small molecule, GFP reconstitution is inhibited accordingly. Using the NHK-drGFP assay, we screened small molecule libraries consisting of 2696 compounds obtained from the NCI Developmental Therapeutics Program (https://dtp.cancer.gov/) on a Tecan Infinite F200 fluorescence microplate reader and identified 12 candidate inhibitors that inhibited protein dislocation by >70% at a concentration of 10 μM. The candidate inhibitors were further independently tested and confirmed in dose-response experiments using different batches of the compounds. Bioinformatic analysis by SwissADME (http://www.swissadme.ch/) revealed that 3 of the 12 candidate inhibitors are pan-assay interference compounds (PAINS). After elimination of the PAINS, the active rate was approximately 0.3% of the total compounds screened. One of the inhibitors, CP26 (1-methyl-4-[(2,3,4,5-tetrachlorocyclopenta-2,4-dien-1-ylidene)methyl]benzene), was selected for further characterization because it exhibited robust activity in the drGFP assay and, to the best of our knowledge, has not previously been reported to exhibit biological activity (Fig. 2A).

### Characterization of CP26 as a dislocation inhibitor

To further characterize CP26, we obtained an independent batch of the compound and determined the dose-dependent effect of CP26 on NHK dislocation in the drGFP assay. CP26 inhibited NHK dislocation with an IC_50_ of 4.45 μM (Fig. 2B). Time-lapse imaging and quantification of NHK-drGFP confirmed CP26 as a dislocation inhibitor (Fig. 2C,D). NHK is a well-established luminal substrate of Hrd1-mediated ERAD that requires the cooperation of nearly two dozen of proteins^24–27^. The ERAD machinery operates largely as an adaptive network, and unique combinations of common components are required for the efficient disposal of individual substrates, a process that is dependent on the structural and biochemical properties of those substrates^26^. We wondered whether CP26 affects the dislocation of CD3δ, a type I membrane substrate that is structurally distinct from NHK^28,29^ (Fig. 1B,C). The inhibitory effects of CP26 on CD3δ dislocation were examined using the CD3δ-drGFP assay, which revealed the decreased potency of CD26 (IC_50_: 34.97 μM) compared to that of NHK-drGFP (IC_50_: 4.45 μM) (Fig. 2B,E). These results suggest that the molecular target of CP26 is essential for dislocation of the luminal substrate NHK and, to a much lesser extent, the membrane substrate CD3δ.

### CP26 enhanced the thermal stability of selected proteins of the Hrd1 complex

**Figure 3:**
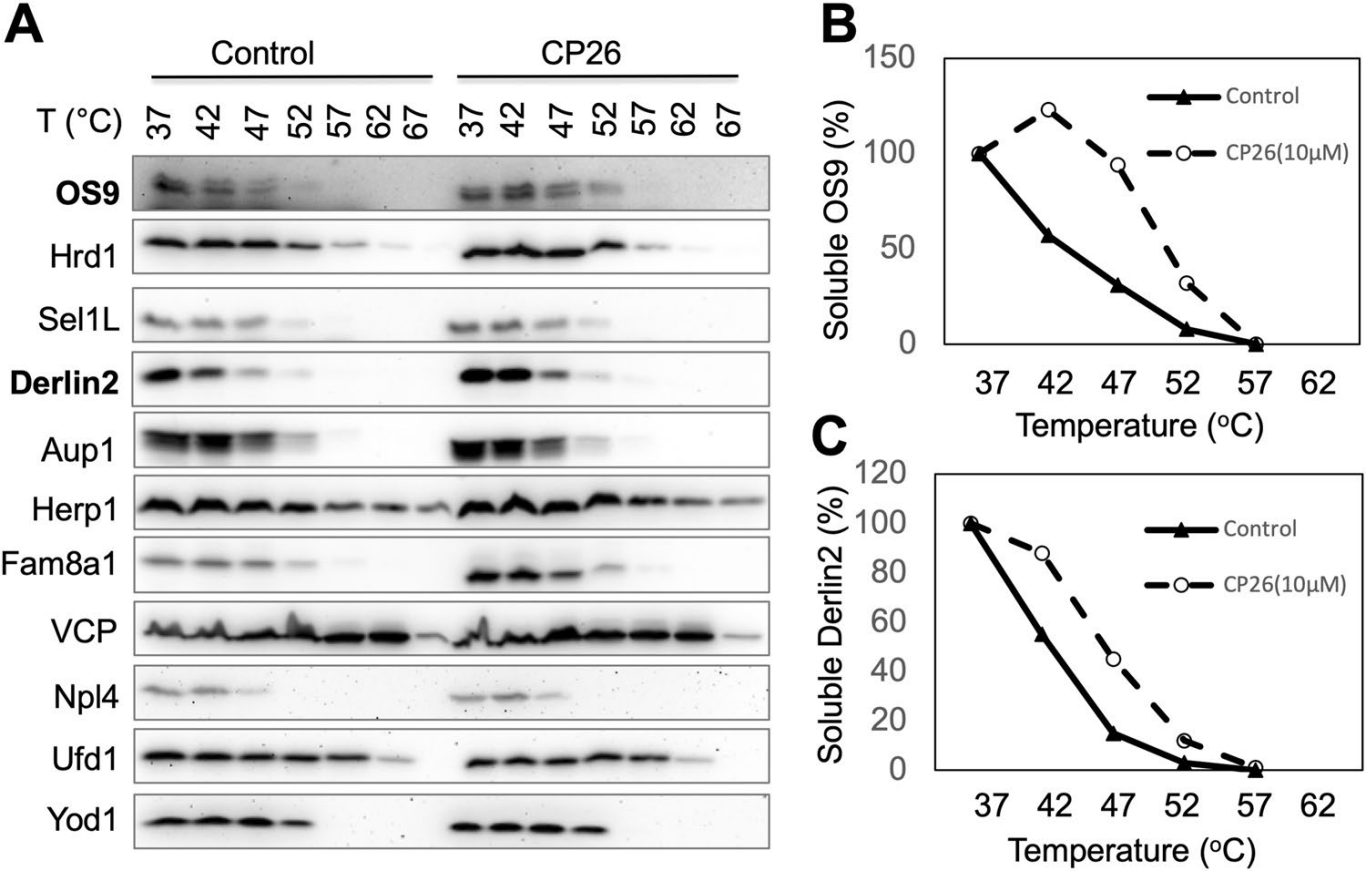
CP26 increases the thermal stability of OS9 and Derlin2. (**A**) The thermal stability of Hrd1 complex proteins in HeLa cells revealed by CETSA. (**B**,**C**) Quantification of the thermal stability of OS9 and Derlin2. The intensities of the bands for OS9 and Derlin2 in (**A**) were quantified.

As a dislocation inhibitor, CP26 is expected to engage its target protein in the Hrd1 complex. Small molecule binding can increase the thermal stability of the target protein. Based on this principle, the cellular thermal shift assay (CETSA) has been established to verify small molecule target engagement^30–33^. We therefore used CETSA to identify the molecular target of CP26. HeLa cells were incubated at increasing temperatures in the presence or absence of CP26. Soluble protein fractions were prepared from the cells and examined by immunoblotting. CP26 selectively increased the thermal stability of Derlin2 and OS9, two of the major components of the Hrd1 complex (Fig. 3). In addition, CP26 increased the steady-state levels and decreased the thermal stability of FAM8A1, another key component of the Hrd1 complex. These results suggest that CP26 targets OS9, Derlin2, FAM8A1, or an unknown protein in the Hrd1 complex, leading to changes in the stability of target-associated proteins. Therefore, CP26 inhibits dislocation by targeting a protein within the Hrd1 complex.

### CP26 inhibited the ubiquitination and degradation of misfolded ER proteins

**Figure 4:**
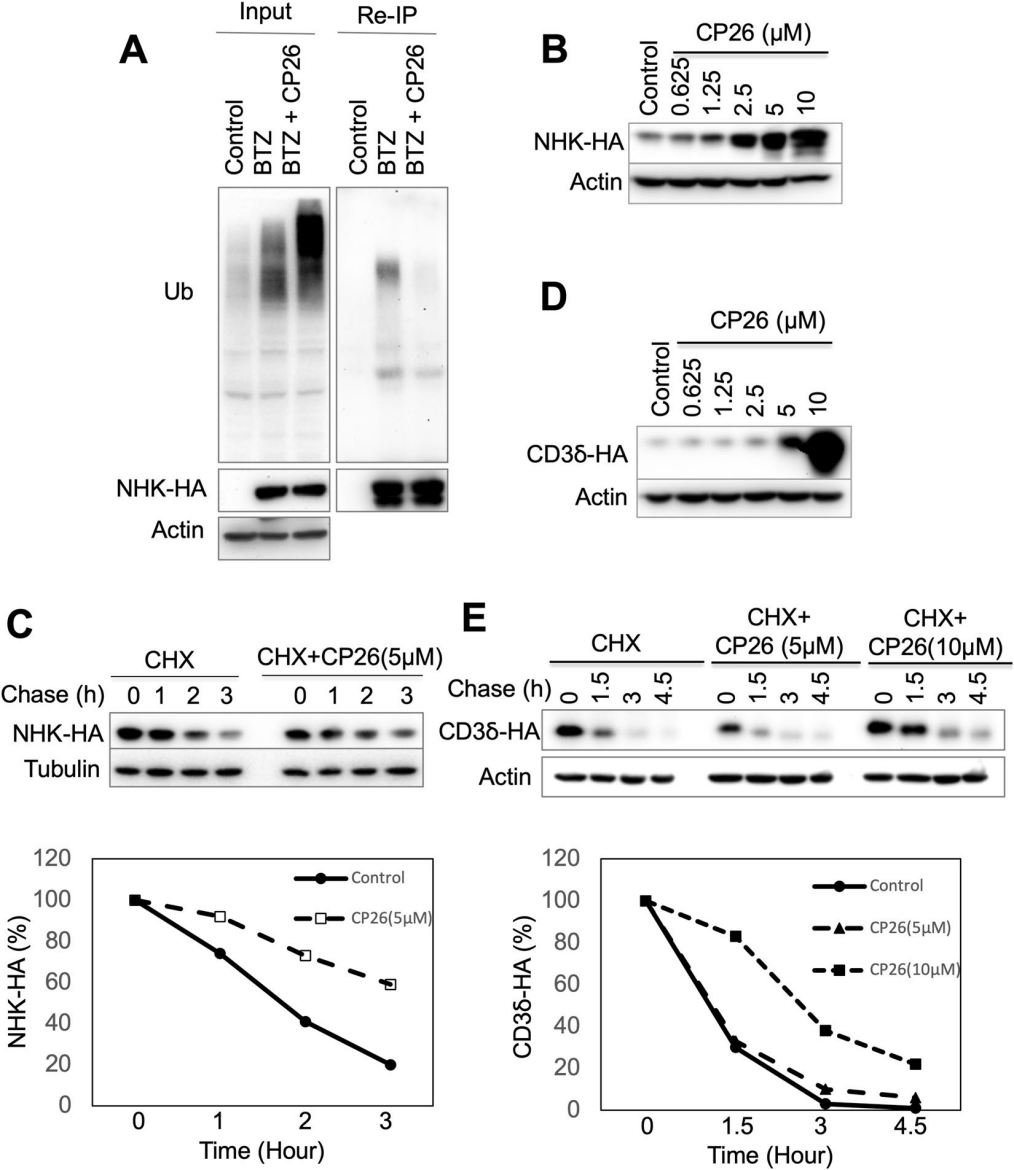
CP26 inhibits NHK ubiquitination and degradation. (**A**) CP26 inhibited NHK ubiquitination in HeLa cells. (**B**,**C**) CP26 stabilized NHK in a dose-dependent manner. (**B**) 293 cells stably expressing NHK-HA were treated with CP26 overnight and then processed for IB. (**C**) NHK degradation was determined by cycloheximide (CHX) chase, and the relative amounts of NHK are shown in the graph. (**D**,**E**) The effects of CP26 on CD3δ degradation were determined as for NHK in (**B**,**C**). 293 cells stably expressing CD3δ tagged with HA were used for these experiments.

Dislocation is prerequisite for the ubiquitination and degradation of ER luminal substrates^16,34^. Therefore, we determined the effects of CP26 on the ubiquitination and degradation of NHK, a well-characterized ER luminal substrate of Hrd1. As shown in Fig. 4, CP26 markedly inhibited NHK ubiquitination compared to that in control cells (Fig. 4A) and stabilized the NHK protein in a dose-dependent manner (Fig. 4B).

**Figure 5:**
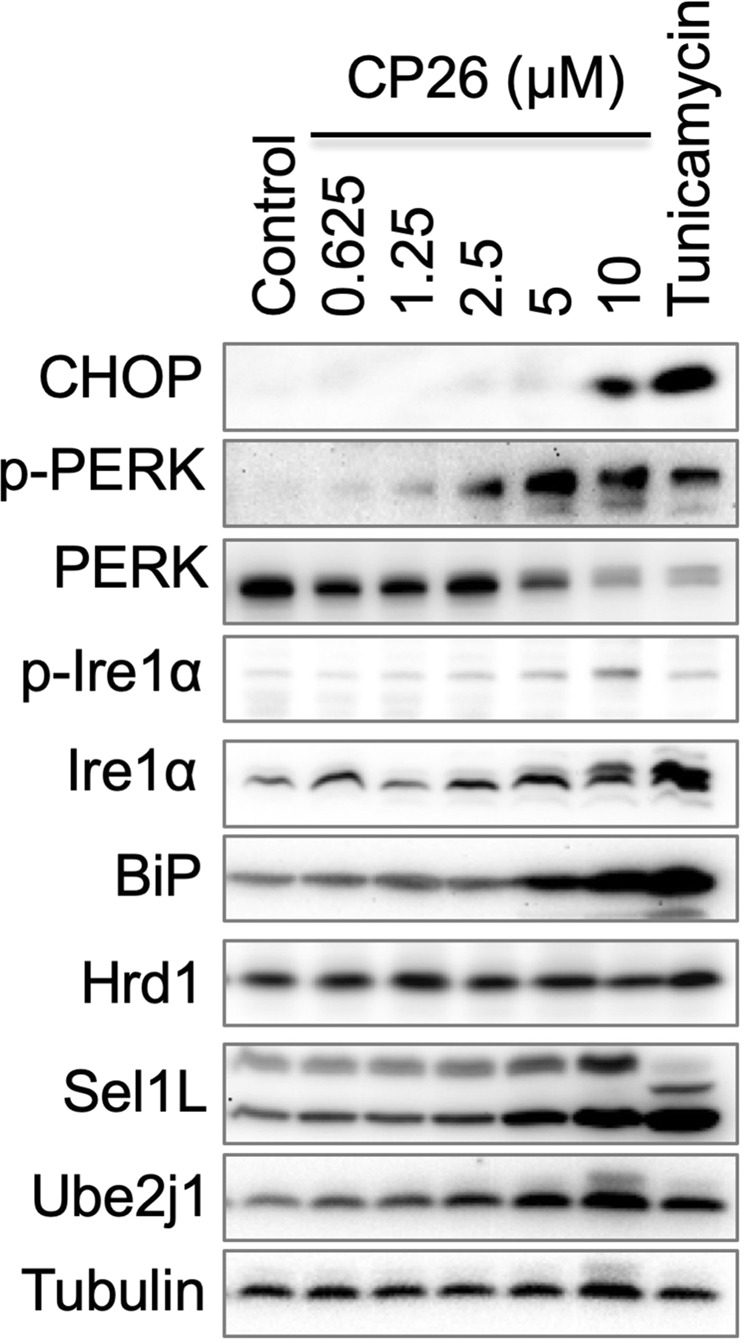
CP26 activates the UPR. HeLa cells were treated with increasing concentrations of CP26 for 24 hours and processed for immunoblotting to examine UPR-associated protein levels. The tunicamycin-induced UPR was used as a positive control.

CP26 inhibited the degradation of NHK (Fig. 4C) in an assay in which cycloheximide (CHX) was used to block protein synthesis, thereby allowing protein degradation to be monitored. CP26 also inhibited CD3δ degradation in the presence of CHX but had a smaller effect compared to its effect on NHK (Fig. 4B–E). This result is consistent with the role of CP26 as a preferential inhibitor of NHK (vs. CD3δ) dislocation (Fig 2B,E). Dislocation inhibition is also expected to cause misfolded protein accumulation in the ER and induce ER stress. Indeed, treatment with CP26 activated the ER stress-induced unfolded protein response (UPR) in a dose-dependent manner^35,36^, as reflected by CP26 dose-dependent increases in the phosphorylation of PERK and Ire1α and upregulation of CHOP and components of the ERAD machinery, such as BiP, Sel1L, and Ube2j1 (Fig. 5). These findings suggest that CP26 engages a target in the Hrd1 complex that is essential for the dislocation of NHK and, to a much lesser extent, CD3δ.

### CP26 inhibits cholera toxin but not ricin activity

**Figure 6:**
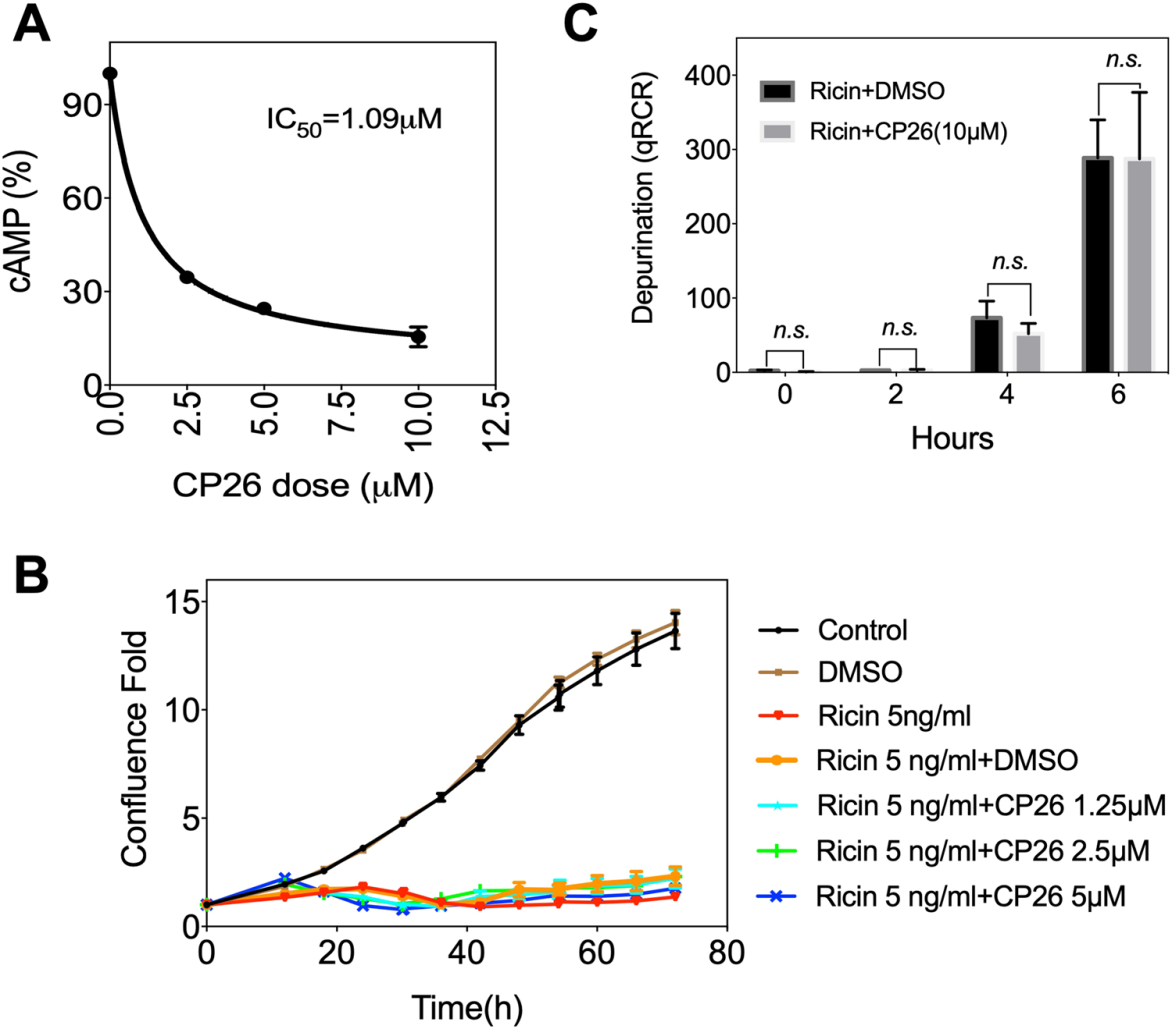
CP26 inhibits the activity of cholera toxin but not ricin. (**A**) CP26 inhibits cholera toxin-induced increases in cAMP in HeLa cells. Data shown are the mean ± SEM, *n* = 3, *p* < 0.0001 vs. cholera toxin alone by ANOVA. (B, C) CP26 does not affect ricin cytotoxicity. (**B**) HeLa cell confluence was recorded in real time and quantified from 0 to 3 days (mean ± SEM, *n* = 3). (**C**) qPCR analysis of ricin-induced depurination (mean ± SEM, *n* = 3). *n.s.:* no significant difference.

Cholera toxin and ricin belong to a group of heterodimeric bacterial and plant toxins containing A and B chain^37–39^. The B chain binds receptors on cell surfaces. The holotoxins enter cells through endocytosis and then undergo retrograde transport to reach the ER lumen^40,41^. In the case of ricin, its A chain and B chain are held together by hydrophobic interactions and a disulfide bond that is reduced in the ER lumen. Protein disulfide isomerase (PDI) is thought to remodel the holotoxin structure to permit reductive cleavage. PDI binds to and unfolds cholera toxin and disassembles its catalytic active A1 chain, which is normally associated with the A2 chain by a disulfide bond. The free enzymatically active A chain of ricin and the A1 chain of CT hijack the dislocation machinery, exit the ER lumen and reach their molecular targets in the cytosol^42–44^. The cholera toxin A chain activates the G protein G_s_α through an ADP-ribosylation reaction, leading to the continuous stimulation of adenylate cyclase to produce cAMP^45,46^. The high cAMP levels activate the cystic fibrosis transmembrane conductance regulator (CFTR), causing a dramatic efflux of ions and water from and between intoxicated enterocytes, leading to watery diarrhoea^45–47^. Therefore, we tested whether CP26 can inhibit cholera toxin activity. We pretreated HeLa cells with CP26 for 10 minutes to ensure inhibition of the dislocation machinery before cholera toxin entered the cells. As shown in Fig. 6A, cholera toxin stimulated an increase in cAMP, and CP26 inhibited this stimulation in a dose-dependent manner (IC_50_: 1.1 μM).

Next, we examined ER dislocation-dependent ricin toxicity. When the ricin A chain is dislocated to the cytosol, it enzymatically inactivates 28S ribosomal RNA (rRNA) via depurinating A4324, resulting in protein synthesis shutdown and cell death by apoptosis^37,43,48^. Therefore, we tested whether inhibiting dislocation by CP26 could alleviate ricin cytotoxicity. Again, we inhibited dislocation by pretreating HeLa cells for 10 minutes, followed by the addition of ricin. Ricin treatment caused a time-dependent cell death that was not affected by CP26 (Fig. 6B). This result was confirmed by quantitative PCR analysis of the ricin-induced depurination of 28S rRNA^48^ (Fig. 6C). Together, these results indicate that CP26 is a dislocation inhibitor and suggest that its molecular target is essential for the dislocation of the cholera toxin A chain but not the ricin A chain.

### CP26 exhibited broad spectrum anti-DENV and anti-ZIKA activity in Huh-7 cells

**Figure 7:**
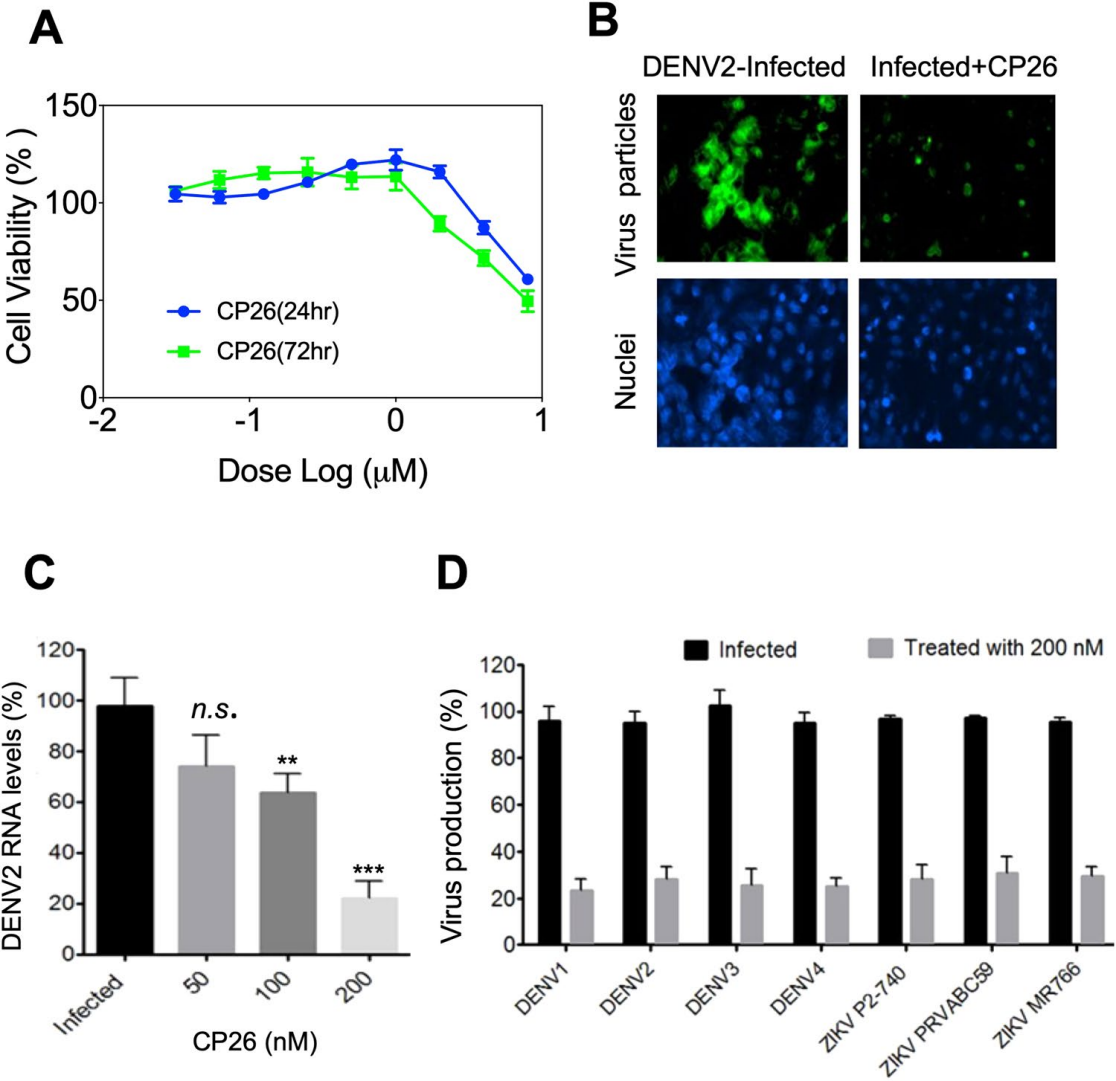
CP26 reduces DENV and ZIKV replications in Huh-7 cells. (**A**) The maximal non-toxic dose of CP26 was determined using Cell Proliferation Reagent WST-1. (**B**) CP26 at 200 nM reduced the number of DENV2 viral particles 48 hrs post-infection, as revealed by immunostaining for the flavivirus group antigen. (**C**) Huh-7 cells were seeded in 12-well plates and infected with DENV2 for 48 hrs at an MOI of 0.1. Virus-infected cells were treated with increasing concentrations of CP26, and viral RNA levels were measured by qPCR. (**D**) Decreased virus production in cells infected by four serotypes of DENV and four strains of ZIKV 48 hrs post-treatment with 200 nM CP26 (ANOVA with Tukey post-test, *n.s.:* no significant difference, **p < 0.01 vs. DMSO control, ***p < 0.001 vs. DMSO control).

Genome-scale RNAi and CRISPR-Cas screens have identified many host factors that are required for DENV, WNV, and ZIKV replication, including proteins in the Hrd1 complex^12–15^. Therefore, we determined whether inhibition of Hrd1-mediated dislocation affects DENV replication. Before conducting the antiviral studies, we determined the maximal non-toxic dose of CP26 to guide the following experiments. CP26 showed no cytotoxic effects against Huh-7 cells at concentrations up to 1 µM (Fig. 7A). We infected Huh-7 cells with DENV2 at an MOI of 0.1 in the absence or presence of 200 nM CP26. CP26 treatment significantly reduced the number of virus particles after 48 hrs of infection, as demonstrated by immunofluorescent staining of flaviviral group antigen (Fig. 7B). Furthermore, CP26 inhibited DENV2 replication in a dose-dependent manner in Huh-7 cells infected with DENV2 (MOI, 0.1), as determined by measuring viral RNA levels using quantitative RT-PCR 48 hrs post-treatment (Fig. 7C). CP26 at 200 nM caused an approximately 80% reduction in viral RNA (Fig. 7C). To determine whether CP26 has broad-spectrum anti-DENV and anti-ZIKV activities, Huh-7 cells were infected with DENV serotypes 1, 2, 3 and 4 and ZIKV stains P2-740, PRVABC59 and MR766 and treated with 200 nM CP26. Virus production was measured by plaque assay 48 hrs post-treatment with CP26. CP26 treatment reduced the production of all DENV serotypes and ZIKV strains examined by nearly 80% (Fig. 7D). These results indicate that CP26 is a broad-spectrum anti-DENV and anti-ZIKA agent *in vitro*.

## Discussion

This study identified CP26 as a novel inhibitor of Hrd1 complex-mediated dislocation using a drGFP-based screen of small molecule libraries. As a dislocation inhibitor, CP26 inhibited ERAD of NHK and CD3δ and induced ER stress. CP26 also inhibited NHK ubiquitination and cholera toxin activity, which are known to require dislocation. CETSAs suggested that the effects of CP26 are due to its direct binding to a target within the Hrd1 complex because CP26 increased the thermal stability of several proteins involved in ER dislocation. Moreover, CP26 exhibited a significant inhibitory effect against DENV and ZIKV replication, which is consistent with recent reports that Hrd1 complex proteins are involved in the replication of these viruses^12–15^. Therefore, the results of the current study suggest that the drGFP assay may be optimized for the high-throughput screening of dislocation inhibitors of the Hrd1 pathway and provides proof-of-principle for targeting the Hrd1 pathway as a strategy to develop broad-spectrum anti-flavivirus drugs. Future studies will be geared towards further defining the molecular target of CP26. Furthermore, CP26 will be used to probe the complex mechanisms of dislocation and how pathogens such as flaviviruses, polyomaviruses, and AB-type toxins hijack dislocation to aid their replication or exert cytotoxicity.

The Hrd1 pathway plays a central role in ER protein quality control. It operates as a large adaptive network, in which the core component, Hrd1, along with a unique combination of other ERAD regulators are involved in the degradation of a specific substrate^26^. NHK is a well-characterized luminal substrate of the Hrd1 pathway. A recent unbiased genome-wide CRISPR-Cas9 screen identified nearly two dozen proteins that act cooperatively in the Hrd1 pathway for NHK identification, delivery to the ER membrane, Sel1L/Hrd1-mediated dislocation to the cytosolic surface of the ER, and ubiquitination/extraction/proteasomal degradation in the cytosol^49^. In contrast, ERAD of another luminal substrate, a non-toxic form of the ricin A chain, requires only approximately half of the ERAD components involved in NHK degradation^49^. This substrate heterogeneity of the Hrd1 ERAD machinery may explain why CP26 differentially inhibited the dislocation/degradation of various substrates, such as NHK, CD3δ, cholera toxin, and ricin. CP26 may target a key component required for the dislocation of NHK, cholera toxin, and, to a much lesser extent, CD3δ, but not the ricin A chain. These differential effects also suggest that CP26 inhibits NHK dislocation by engaging a specific target and not by general impairment of the ERAD machinery.

Previous studies using genome-wide siRNA and CRISPR-Cas9 screening identified several components of the Hrd1 ERAD complex as essential host factors required for the replication of WNV, DENV and/or ZIKA^12–15^. Thus, it is not surprising that CP26, an inhibitor of the Hrd1-mediated ERAD pathway, possesses potent anti-DENV and anti-ZIKV activity. However, our data do not provide a precise mechanism by which CP26 inhibits the replication of these viruses. The answer to this question will rely on future investigation that strengthens our understanding of the role of Hrd1 complex proteins in the flavivirus life cycle, which may lead to the identification of the molecular target of CP26, or vice versa. We speculate that CP26 may inhibit DENV and ZIKV replication by interfering with intracellular events during their replication, such as protein quality control of viral proteins, viral particle assembly, and/or trafficking. The antiviral activity of CP26 may not be limited to flaviviruses. For example, polyomavirus translocates across the ER membrane to reach the cytosol by hijacking the dislocation pathway as part of its infection strategy^39,50–52^. A previous study showed that inhibiting specific components of the ERAD pathway using dominant negatives or RNAi blocked polyomavirus infection^53^. In addition, CP26 may be used to inhibit the toxicity of cholera toxin as a new approach to treat diarrhoea caused by *Vibrio cholerae* infection.

Paradoxically, both the inhibition of dislocation by CP26 and flavivirus infection induce ER stress, a condition that might help an active virus replicate or lead to the death of infected cells. For example, DENV infection stimulates the UPR for optimal translation of the viral proteins in the ER to enhance viral replication^54,55^. UPR induction improves conditions in the ER for viral replication by increasing the ER volume and upregulating protein folding chaperones^56^. A recent study showed that ZIKV infection initiates ER stress and triggers the UPR in human cerebral cortices^57^. The disorganized UPR in cerebral cortices due to ZIKV infection led to a decrease in the production of neuronal projections, causing microcephaly in newborns. Thus, pharmacological inhibition of the UPR inhibited the pathogenesis of ZIKV infection and reduced the incidence of microcephaly^57^. The WNV-induced UPR activates PERK, which in turn phosphorylates elF2α, leading to upregulation of the transcription factor CHOP, causing premature neuronal cell death^58^. Similarly, the JEV-induced UPR also causes apoptosis of neuronal cells via a CHOP-mediated pathway^59^. An important question is why CP26 induces ER stress and does not help but rather inhibits DENV and ZIKV replication. A likely reason is that CP26 treatment activates only the protective adaptive UPR, while the inhibition of viral replication eliminates virus-caused lethality due to the apoptotic UPR. For those cells infected before CP26 treatment, CP26 may activate the apoptotic UPR by enhancing the virus-induced UPR, which would eradicate the infected cells while simultaneously inhibiting viral replication in adjacent cells. In fact, cell death is a well-known defence mechanism to reduce viral replication, and some viruses interfere with this mechanism as a strategy to maintain their replication^60^. Another possibility is that the Hrd1 complex is involved in virus replication and that inhibition of its function by CP26 therefore leads to its anti-viral activity. In addition, the concentration of CP26 required to efficiently inhibit DENV and ZIKV replication is much lower than its maximum tolerated dose; therefore, ER stress is not induced by the doses used to inhibit viral replication. Moreover, small molecule inhibitors of the key components of dislocation pathways, such as p97/VCP, Npl4, and BiP, are well tolerated in mice^61–63^. Therefore, inhibiting dislocation may be a feasible approach for the development of anti-flaviviral drugs.

## Conclusion

This study demonstrates for the first time that a small molecule inhibitor of Hrd1 complex-mediated dislocation potently inhibits DENV and ZKV infection in cells. The role of the Hrd1 complex in flavivirus infection remains elusive, and the molecular target of CP26 is not known. Thus, further studies are required to explore the physical and functional interactions among flaviviral proteins and Hrd1 complex proteins and to identify the molecular target of CP26, which will help us understand how the Hrd1 complex regulates flavivirus replication and how CP26 exerts its antiviral activity.

## Materials and Methods

### Viruses, cells, antibodies, chemicals and other materials

Ricin and all viruses were obtained from www.beiresources.org. Cholera toxin (C8052) and tunicamycin (T7765) were purchased from Sigma-Aldrich (St. Louis, MO, USA). C6/36 mosquito cells (CRL-1660), Vero cells (CCL-81) and HeLa cells (CCL-2) were obtained from ATCC (Manassas, VA, USA). Huh-7 cells were provided by Dr. Hongbing Wang. All cells were cultured in complete Dulbecco’s modified Eagle’s medium (DMEM) supplemented with 10% foetal bovine serum (FBS) as growth medium or 2% FBS as maintenance medium. Bortezomib (BTZ), cycloheximide (CHX), and tunicamycin were purchased from Sigma-Aldrich (St. Louis, MO, USA) or Thermo Fisher Scientific (Waltham, MA, USA). The sources of the antibodies are as follows: antibodies against A1AT, HA, actin, Aup1, Derlin2, and Sel1L (Sigma-Aldrich, St. Louis, MO, USA); antibodies against CHOP, PERK, p-PERK, Ire1𝛼, and p-Ire1𝛼 (Cell Signaling, Danvers, MA, USA); antibodies against BiP and VCP (BD Biosciences, San Jose, CA, USA); antibodies against Hrd1 and OS9 (Abcam, Cambridge, MA, USA); antibodies against Ube2j1 and Npl4 (Santa Cruz Biotechnology, Dallas, TX, USA); antibody against Herp1 (OriGene, Rockville, MD, USA); antibody against Fam8A1 (Abgent, San Diego, CA, USA); antibody against Ufd1 (Bethyl, Montgomery, TX, USA); and antibody against Yod1 (Invitrogen, Carlsbad, CA, USA).

### Identification of small molecule inhibitors of ER protein dislocation

We previously established a HeLa cell line stably expressing a dislocation-induced reconstituted GFP (drGFP) reporter to monitor and quantify NHK dislocation from the ER (NHK-drGFP)^21^. The NHK-drGFP assay was used to screen small molecule libraries (2696 compounds) obtained from the NCI Developmental Therapeutics Program (https://dtp.cancer.gov/). HeLa cells expressing NHK-drGFP and CD3δ-drGFP reporter^21^ were cultured in 96-well black and clear-bottom plates (3603, Costar) at 2 × 10^4^/well overnight. Cells were then treated with the proteasome inhibitor bortezomib (BTZ, 1 µM) alone or with compound (10 μM) for 4 hours. drGFP fluorescence intensities before and after treatment were measured on a Tecan F200 Pro multimode microplate reader using excitation = 488 nm and emission = 525 nm. Compounds exhibiting >70% inhibition were subjected to auto-green fluorescence measurement, and fluorescent compounds were eliminated. The remaining compounds were retested by NHK-drGFP assay and denoted dislocation inhibitors when confirmed. All dislocation inhibitors were further tested independently in an InCell Analyzer 2200 wide-field imaging system at NCATS using different batches of compounds at a concentration of 10 μM.

### Live-cell imaging and quantification of drGFP

NHK drGFP HeLa cells were seeded in 96-well plates. After overnight culture, the cells were washed once with PBS and then treated with BTZ (1 μM) alone or BTZ (1 μM) together with CP26 (1, 5, or 10 μM). Live-cell images were acquired immediately after the addition of the inhibitors and every 30 min thereafter on an IncuCyte Live-Cell Analysis System with a 20× objective lens (Sartorius, Ann Arbor, MI, USA).

### Cellar thermal shift assay (CETSA)

CETSA was used to determine the effects of CP26 on the thermal stability of Hrd1 complex proteins following a previously reported protocol^30,33^. Briefly, HeLa cells were treated with 10 μM CP26 at 37 °C for 2 h, and DMSO-treated cells were used as a control. After treatment, cells were lysed in 0.4% NP-40. Multiple aliquots of cell lysate were heated at increasing temperatures for 3 minutes. After cooling, the samples were centrifuged at 20,000 × g for 20 min to separate soluble fractions from precipitated proteins. The soluble fractions were incubated with 4 × SDS sample buffer for 30 minutes at 37 °C to minimize oligomerization of transmembrane proteins before processing for immunoblotting (IB) following a previously published procedure^29^. The major candidate target proteins of the Hrd1 complex examined were Hrd1, Sel1L, OS9, Derlin2, Aup1, Herp1, Fam8a1, Yod1, VCP, Ufd1, and Npl4.

### Analysis of NHK ubiquitination in cells

NHK ubiquitination was analysed as previously reported^21^. Briefly, HeLa cells expressing NHK-HA were treated with bortezomib (BTZ, 1 μM) or BTZ + CP26 (10 μM) to prevent degradation of ubiquitinated NHK. After treatment, the cells were lysed under native conditions followed by immunoprecipitation (IP) with anti-HA antibody-conjugated beads. To remove any protein that may associate with NHK-HA, the immunoprecipitates were denatured with 2% SDS, and the beads were removed by centrifugation. The supernatants were then diluted 20 times in native lysis buffer from which NHK-HA was re-immunoprecipitated (re-IP) followed by IB for HA and ubiquitin.

### Cycloheximide chase

Previously described HeLa cells stably expressing HA-tagged NHK or CD3δ were seeded (2 × 10^5^ cells/well) in a 12-well plate and cultured overnight^21,64^. Cycloheximide chase was performed as previously reported^29^. Briefly, cells were incubated with the translational inhibitor cycloheximide alone or with CP26 for the indicated time (Fig. 4C,E), and cells were then harvested and processed for IB.

### Cholera toxin and ricin toxicity assay

#### Measuring cholera toxin-stimulated cAMP production

HeLa cells were seeded in a 96-well plate at a final volume of 100 μL containing 10^4^ cells per well. After overnight culture, cells were pretreated with different doses of CP26 (2.5, 5, or 10 μM) or DMSO for 10 min. In the presence of CP26, cells were challenged for 30 min with cholera toxin (11.8 nM). The cAMP level was measured from the collected lysates using a Cyclic AMP XP chemiluminescent assay kit (Cell Signaling Technologies, 8019) following the manufacturer’s instructions. The cAMP levels were measured using a Tecan F200 Pro multimode microplate reader.

#### Measuring ricin cytotoxicity and depurination

HeLa cells cultured in 96-well dishes were pretreated with CP26 (1.5, 2.5, or 5 μM) or DMSO for 10 minutes, and then ricin (5 ng/ml) was added to the cells. Controls included cells treated with ricin or DMSO. Cell confluence was used to measure cell viability and measured in real time with an IncuCyte Live-Cell Analysis System. The effects of CP26 on the ricin-catalysed depurination of 28S rRNA were determined by quantitative RT-PCR (qRT-PCR) following a previously reported protocol^48^. This qRT-PCR assay relied on an inherent property of reverse transcriptase in which it incorporates a deoxyadenosine at the position opposite the abasic site. The resultant T to A transversion on the cDNA strand is detected by sequence-specific primers. Briefly, HeLa cells were seeded at 3 × 10^5^ per well in 6-well plates and allowed to attach overnight. After incubation with 10 μΜ CP26 (or 0.05% DMSO) for 1 h at 37 °C, cells were challenged with 5 ng/ml ricin for 0–6 h. RNA was prepared with a High Pure RNA Isolation Kit (Roche) following the manufacturer’s instructions. RNA was reverse-transcribed with a PhotoScript^®^ First Strand cDNA Synthesis Kit (New England Biolabs). The primer sets were designed as previously reported^48^. The forward and reverse primers to amplify the total 28S rRNA were 5′-GAT GTC GGC TCT TCC TAT CAT TGT-3′ and 5′-CCA GCT CAC GTT CCC TAT TAG TG-3′. The forward and reverse primers used to specifically detect the RPI-induced change in sequence (depurination) were 5′-TGC CAT GGT AAT CCT GCT CAG TA-3′ and 5′-TCT GAA CCT GCG GTT CCA CA-3′. Real-time PCR was performed in a CFX96^TM^ Real Time System (Bio-Rad) using iQ^TM^ SYBR^®^ Green Supermix. Each cDNA sample was run in triplicate with each primer pair. Bio-Rad software was used to obtain threshold cycle (CT) numbers from the real-time PCR runs. The ΔCT between the target (depurinated rRNA) and endogenous reference (total rRNA) was determined for a given sample. Next, the ΔΔCT, in which the ΔCT for a calibration sample was subtracted from the ΔCT of the test sample, was established. The calibration samples were not treated with ricin (the 0 h point). Then, the 2^(−ΔΔCT)^ value of each test sample was calculated.

### Maximal non-toxic dose (MNTD)

Huh-7 cells (1 × 10^4^ cells per well of a 96-well plate) were treated with increasing concentrations (31.25 nM to 8 µM) of CP26 for 24 and 72 hrs. The cytotoxic effects of CP26 were determined using Cell Proliferation Reagent WST-1 (Sigma-Aldrich, St. Louis, MO, USA). The percentage of cell viability was calculated as follows: 100% − (absorbance of treated cells/absorbance of untreated cells) × 100%.

### Anti-DENV and anti-ZIKV activity of CP26

Dengue virus serotypes and ZIKV strains were propagated by a single passage. The viral titres were determined by plaque assay using Vero cells following our previously reported procedure^65,66^. To determine the antiviral activity of CP26 using plaque assays, Huh-7 cells were seeded in 12-well plates (3 × 10^5^ cell/well) for 24 hrs. Then, the cells were infected with either DENV2 or ZIKV at an MOI of 1 and treated with the compound. The culture supernatant was collected at 48 hrs post-infection, and a plaque assay was performed to measure virus titre as we previously described^65,66^. Briefly, 10-fold serial diluted supernatant of cultured virus-infected cells was added to fresh Vero cells grown in 6-well plates (5 × 10^5^ cells) and incubated for 1 hr at 37 °C. The cells were overlaid with DMEM (maintenance medium) containing 0.5% agarose. Viral plaques were stained with crystal violet dye after 5 days of incubation. Virus titres were calculated according to the following formula: titre (pfu/ml) = number of plaques/volume of the diluted virus added to the well × dilution factor of the virus used to infect the well in which the plaques were counted. To determine the antiviral activity of CP26 using quantitative RT-PCR, Huh-7 cells were plated in 24-well plates (in triplicate for each condition), infected at an MOI of 0.1, and treated with CP26. Viral RNA was purified using a QIAamp viral RNA extraction mini kit (Qiagen, USA) at 48 hpi. After the RT reaction, qPCR was performed using the DENV2 forward primer (5′ACATCTCAAGTGCAGGCTGA3′) and reverse primer (5′GTCTCCGAATGGAGGTTCTG3′), and viral RNA levels were normalized to GAPDH RNA levels. To determine the antiviral activity of CP26 using immunostaining, Huh-7 cells were seeded on cover slips in a 12-well plate and infected with DENV-2 at an MOI of 0.1. Cells were then treated with 200 nM CP26 for 48 hrs, followed by fixation using 4% paraformaldehyde. Viral particles were stained using anti-flavivirus group antigen antibody (clone D1-4G2-4-15, EMD Millipore, Burlington, MA, USA) at 1:1,000 in blocking buffer for 1 h, washed 3 times and incubated with Alexa 488-conjugated goat anti-mouse immunoglobulin G (IgG) (Life Technologies, Carlsbad, CA, USA) and DAPI (Insitus, Albuquerque, NM, USA) for 30 min. After 3 washes with PBS, cells were visualized using a Nikon TRE fluorescence microscope.

### Statistical analysis

All data are presented as the mean ± SEM. ANOVA was performed to analyse the effects of ricin-stimulated depurination and DENV2 production. Probability values < 0.05 indicated statistical significance.

## Supplementary information

Original blot images

## Data Availability

No datasets were generated or analysed during the current study.

## References

[1] Bhatt S (2013). The global distribution and burden of dengue. Nature.

[2] Baud D, Gubler DJ, Schaub B, Lanteri MC, Musso D (2017). An update on Zika virus infection. Lancet.

[3] Pierson TC, Diamond MS (2018). The emergence of Zika virus and its new clinical syndromes. Nature.

[4] Shan C, Xie X, Shi PY (2018). Zika Virus Vaccine: Progress and Challenges. Cell Host Microbe.

[5] Heymann DL (2016). Zika virus and microcephaly: why is this situation a PHEIC. Lancet.

[6] Fatima K, Syed NI (2018). Dengvaxia controversy: impact on vaccine hesitancy. J Glob Health.

[7] Gotuzzo E, Yactayo S, Córdova E (2013). Efficacy and duration of immunity after yellow fever vaccination: systematic review on the need for a booster every 10 years. Am J Trop Med Hyg.

[8] Rothan HA, Bidokhti MRM, Byrareddy SN (2018). Current concerns and perspectives on Zika virus co-infection with arboviruses and HIV. J Autoimmun.

[9] Boldescu V, Behnam MAM, Vasilakis N, Klein CD (2017). Broad-spectrum agents for flaviviral infections: dengue, Zika and beyond. Nat Rev Drug Discov.

[10] Plummer E (2015). Dengue Virus Evolution under a Host-Targeted Antiviral. J Virol.

[11] Barrows NJ (2016). A Screen of FDA-Approved Drugs for Inhibitors of Zika Virus Infection. Cell Host Microbe.

[12] Krishnan MN (2008). RNA interference screen for human genes associated with West Nile virus infection. Nature.

[13] Mairiang D (2013). Identification of new protein interactions between dengue fever virus and its hosts, human and mosquito. PLoS One.

[14] Ma H (2015). A CRISPR-Based Screen Identifies Genes Essential for West-Nile-Virus-Induced Cell Death. Cell Rep.

[15] Scaturro P (2018). An orthogonal proteomic survey uncovers novel Zika virus host factors. Nature.

[16] Vembar SS, Brodsky JL (2008). One step at a time: endoplasmic reticulum-associated degradation. Nat Rev Mol Cell Biol.

[17] Tsai B, Ye Y, Rapoport TA (2002). Retro-translocation of proteins from the endoplasmic reticulum into the cytosol. Nat Rev Mol Cell Biol.

[18] Hebert DN, Molinari M (2007). In and out of the ER: protein folding, quality control, degradation, and related human diseases. Physiol Rev.

[19] Chen Z, Du S, Fang S (2012). gp78: a multifaceted ubiquitin ligase that integrates a unique protein degradation pathway from the endoplasmic reticulum. Curr Protein Pept Sci.

[20] Molinari M, Hebert DN (2015). Glycoprotein maturation and quality control. Semin Cell Dev Biol.

[21] Zhong Y, Fang S (2012). Live cell imaging of protein dislocation from the endoplasmic reticulum. J Biol Chem.

[22] Cabantous S, Terwilliger TC, Waldo GS (2005). Protein tagging and detection with engineered self-assembling fragments of green fluorescent protein. Nat Biotechnol.

[23] Cabantous S, Waldo GS (2006). *In vivo* and *in vitro* protein solubility assays using split GFP. Nat Methods.

[24] Nagasawa K, Higashi T, Hosokawa N, Kaufman RJ, Nagata K (2007). Simultaneous induction of the four subunits of the TRAP complex by ER stress accelerates ER degradation. EMBO Rep.

[25] Bernasconi R, Pertel T, Luban J, Molinari M (2008). A dual task for the Xbp1-responsive OS-9 variants in the mammalian endoplasmic reticulum: inhibiting secretion of misfolded protein conformers and enhancing their disposal. J Biol Chem.

[26] Christianson JC (2012). Defining human ERAD networks through an integrative mapping strategy. Nat Cell Biol.

[27] Zhong Y (2015). Identification of ERAD components essential for dislocation of the null Hong Kong variant of α-1-antitrypsin (NHK). Biochem Biophys Res Commun.

[28] Tiwari S, Weissman AM (2001). Endoplasmic reticulum (ER)-associated degradation of T cell receptor subunits. Involvement of ER-associated ubiquitin-conjugating enzymes (E2s). J Biol Chem.

[29] Fang S (2001). The tumor autocrine motility factor receptor, gp78, is a ubiquitin protein ligase implicated in degradation from the endoplasmic reticulum. Proc Natl Acad Sci USA.

[30] Martinez Molina D (2013). Monitoring drug target engagement in cells and tissues using the cellular thermal shift assay. Science.

[31] Martinez Molina D, Nordlund P (2016). The Cellular Thermal Shift Assay: A Novel Biophysical Assay for *In Situ* Drug Target Engagement and Mechanistic Biomarker Studies. Annu Rev Pharmacol Toxicol.

[32] Martinez NJ (2018). A widely-applicable high-throughput cellular thermal shift assay (CETSA) using split Nano Luciferase. Sci Rep.

[33] Reinhard FB (2015). Thermal proteome profiling monitors ligand interactions with cellular membrane proteins. Nat Methods.

[34] Christianson JC, Ye Y (2014). Cleaning up in the endoplasmic reticulum: ubiquitin in charge. Nat Struct Mol Biol.

[35] Wang S, Kaufman RJ (2012). The impact of the unfolded protein response on human disease. J Cell Biol.

[36] Wang M, Kaufman RJ (2014). The impact of the endoplasmic reticulum protein-folding environment on cancer development. Nat Rev Cancer.

[37] Audi J, Belson M, Patel M, Schier J, Osterloh J (2005). Ricin poisoning: a comprehensive review. JAMA.

[38] Wahome PG, Robertus JD, Mantis NJ (2012). Small-molecule inhibitors of ricin and Shiga toxins. Curr Top Microbiol Immunol.

[39] Inoue T, Tsai B (2013). How viruses use the endoplasmic reticulum for entry, replication, and assembly. Cold Spring Harb Perspect Biol.

[40] Tsai B, Rodighiero C, Lencer WI, Rapoport TA (2001). Protein disulfide isomerase acts as a redox-dependent chaperone to unfold cholera toxin. Cell.

[41] Spooner RA (2004). Protein disulphide-isomerase reduces ricin to its A and B chains in the endoplasmic reticulum. Biochem J.

[42] Bernardi KM (2010). The E3 ubiquitin ligases Hrd1 and gp78 bind to and promote cholera toxin retro-translocation. Mol Biol Cell.

[43] Spooner RA, Lord JM (2015). Ricin trafficking in cells. Toxins (Basel).

[44] Williams JM, Tsai B (2016). Intracellular trafficking of bacterial toxins. Curr Opin Cell Biol.

[45] Simon NC, Aktories K, Barbieri JT (2014). Novel bacterial ADP-ribosylating toxins: structure and function. Nat Rev Microbiol.

[46] Das B (2016). Molecular evolution and functional divergence of Vibrio cholerae. Curr Opin Infect Dis.

[47] Guichard A (2013). Cholera toxin disrupts barrier function by inhibiting exocyst-mediated trafficking of host proteins to intestinal cell junctions. Cell Host Microbe.

[48] Melchior WB, Tolleson WH (2010). A functional quantitative polymerase chain reaction assay for ricin, Shiga toxin, and related ribosome-inactivating proteins. Anal Biochem.

[49] Leto DE (2019). Genome-wide CRISPR Analysis Identifies Substrate-Specific Conjugation Modules in ER-Associated Degradation. Mol Cell.

[50] Byun H, Gou Y, Zook A, Lozano MM, Dudley JP (2014). ERAD and how viruses exploit it. Front Microbiol.

[51] Dupzyk A, Tsai B (2016). How Polyomaviruses Exploit the ERAD Machinery to Cause Infection. Viruses.

[52] Ravindran MS, Bagchi P, Cunningham CN, Tsai B (2016). Opportunistic intruders: how viruses orchestrate ER functions to infect cells. Nat Rev Microbiol.

[53] Lilley BN, Gilbert JM, Ploegh HL, Benjamin TL (2006). Murine polyomavirus requires the endoplasmic reticulum protein Derlin-2 to initiate infection. J Virol.

[54] Sepúlveda-Salinas KJ, Ramos-Castañeda J (2017). Participation of dengue virus NS4B protein in the modulation of immune effectors dependent on ER stress in insect cells. Cell Stress Chaperones.

[55] Perera N, Miller JL, Zitzmann N (2017). The role of the unfolded protein response in dengue virus pathogenesis. Cell Microbiol.

[56] Lewy TG, Grabowski JM, Bloom ME (2017). BiP: Master Regulator of the Unfolded Protein Response and Crucial Factor in Flavivirus Biology. Yale J Biol Med.

[57] Gladwyn-Ng I (2018). Stress-induced unfolded protein response contributes to Zika virus-associated microcephaly. Nat Neurosci.

[58] Medigeshi GR (2007). West Nile virus infection activates the unfolded protein response, leading to CHOP induction and apoptosis. J Virol.

[59] Su HL, Liao CL, Lin YL (2002). Japanese encephalitis virus infection initiates endoplasmic reticulum stress and an unfolded protein response. J Virol.

[60] Upton JW, Chan FK (2014). Staying alive: cell death in antiviral immunity. Mol Cell.

[61] Anderson DJ (2015). Targeting the AAA ATPase p97 as an Approach to Treat Cancer through Disruption of Protein Homeostasis. Cancer Cell.

[62] Cerezo M (2016). Compounds Triggering ER Stress Exert Anti-Melanoma Effects and Overcome BRAF Inhibitor Resistance. Cancer Cell.

[63] Skrott Z (2017). Alcohol-abuse drug disulfiram targets cancer via p97 segregase adaptor NPL4. Nature.

[64] Zhong Y (2011). Importin beta interacts with the endoplasmic reticulum-associated degradation machinery and promotes ubiquitination and degradation of mutant alpha1-antitrypsin. J Biol Chem.

[65] Rothan HA, Bahrani H, Shankar EM, Rahman NA, Yusof R (2014). Inhibitory effects of a peptide-fusion protein (Latarcin-PAP1-Thanatin) against chikungunya virus. Antiviral Res.

[66] Rothan HA (2016). Mefenamic acid in combination with ribavirin shows significant effects in reducing chikungunya virus infection *in vitro* and *in vivo*. Antiviral Res.

