# Supplementary material for: A small molecule inhibitor of ER-to-cytosol protein dislocation exhibits anti-dengue and anti-Zika virus activity
Source: Sci Rep. 2019 Jul 29;9:10901. doi: 10.1038/s41598-019-47532-7 (PMC6662757; doi:10.1038/s41598-019-47532-7)
Supplement: Supplementary file 1 — Original blot images [file 41598_2019_47532_MOESM1_ESM.pdf]

Supplemental Information

A small molecule inhibitor of ER-to-cytosol protein dislocation exhibits anti-dengue and anti-Zika virus activity

Jingjing Ruan[1, 2], Hussin A. Rothan[2#], Yongwang Zhong[2], Wenjing Yan[2], Mark J. Henderson[3], Feihu Chen[1]*, Shengyun Fang[2]*

[1]Anhui Medical University School of Pharmacy, Hefei, Anhui 230032, China; [2]Center for Biomedical Engineering and Technology, Department of Physiology, Department of Biochemistry and Molecular Biology, University of Maryland School of Medicine, Baltimore, MD 21201; [3]National Center for Advancing Translational Sciences, National Institutes of Health, Rockville, MD 20850.

*Correspondence to: FC: cfhchina@qq.com, or SF: sfang@som.umaryland.edu
# Current address: Department of Biology, College of Arts and Sciences, Georgia State University, Atlanta, GA 30303.

Figure 3

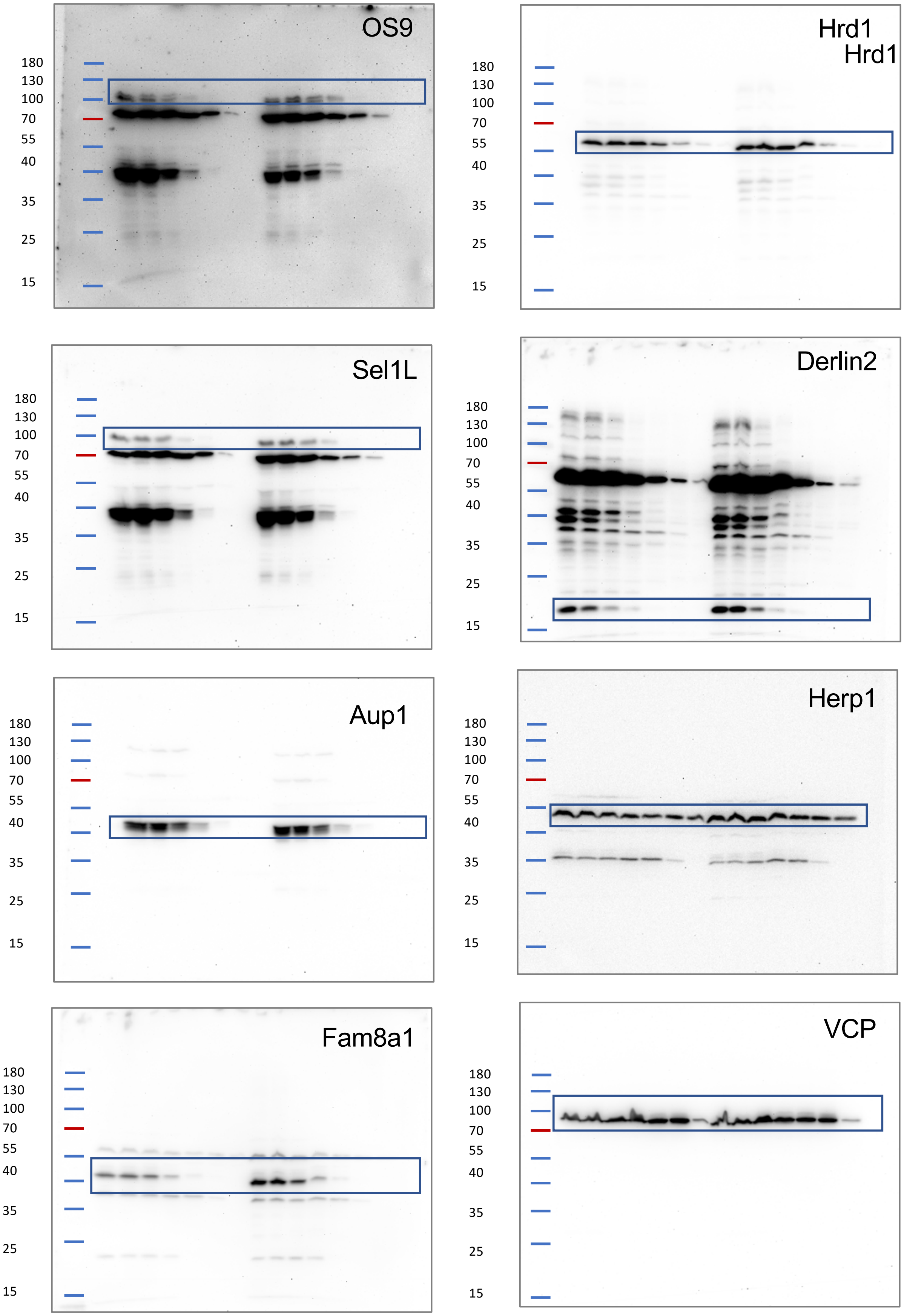
OS9
180
130
100
70
55
40
35
25
15
Hrd1
Hrd1
180
130
100
70
55
40
35
25
15
Sel1L
180
130
100
70
55
40
35
25
15
Derlin2
180
130
100
70
55
40
35
25
15
Aup1
180
130
100
70
55
40
35
25
15
Herp1
180
130
100
70
55
40
35
25
15
Fam8a1
180
130
100
70
55
40
35
25
15
VCP
180
130
100
70
55
40
35
25
15

Figure 3

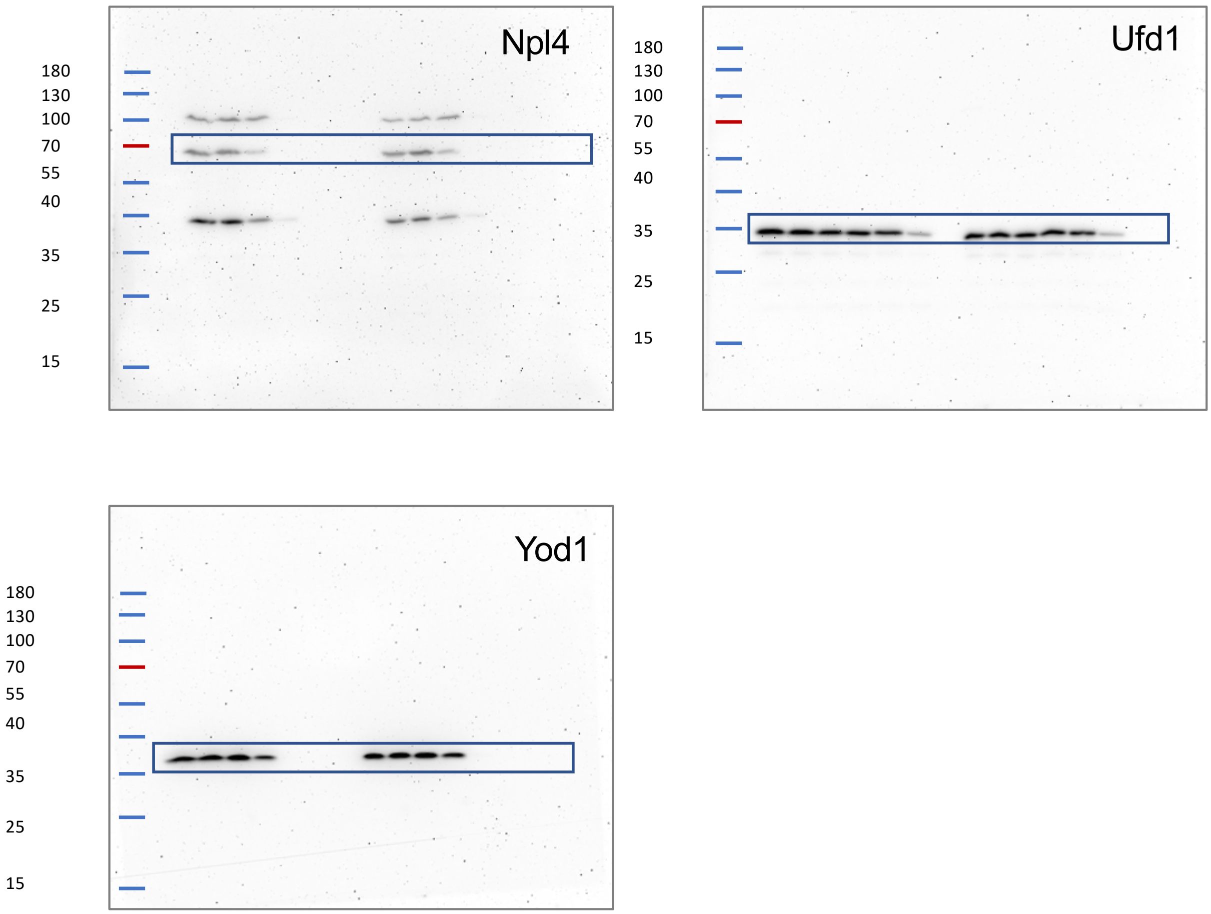

Npl4
180
130
100
70
55
40
35
25
15
Ufd1
180
130
100
70
55
40
35
25
15
Yod1
180
130
100
70
55
40
35
25
15

Figure 4A

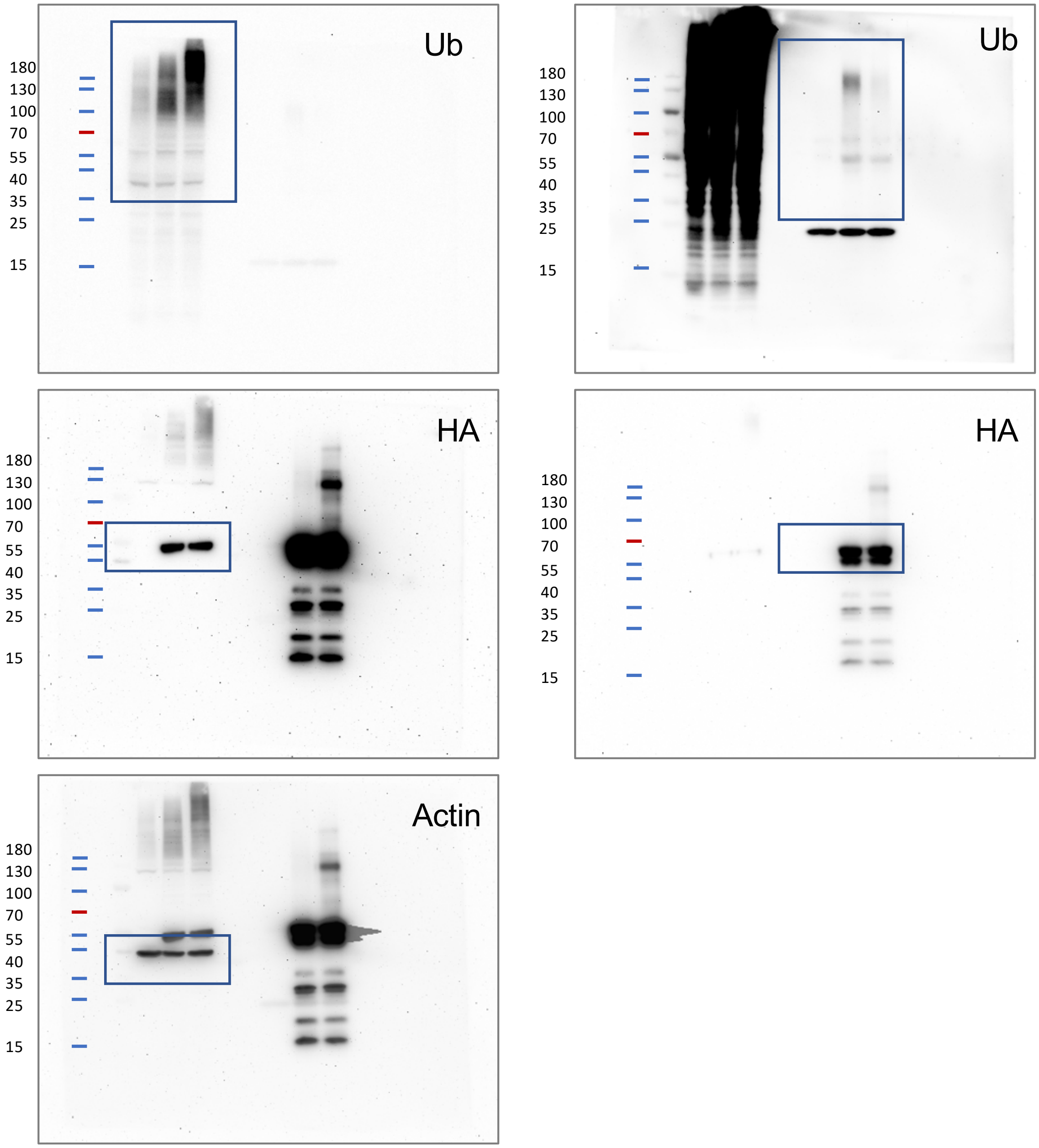

Ub
180
130
100
70
55
40
35
25
15
Ub
180
130
100
70
55
40
35
25
15
HA
180
130
100
70
55
40
35
25
15
HA
180
130
100
70
55
40
35
25
15
Actin
180
130
100
70
55
40
35
25
15

Figure 4B

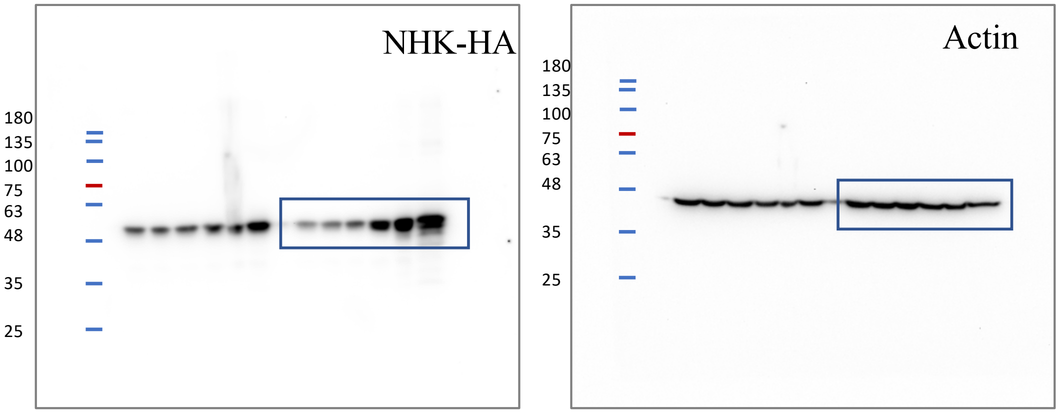

Figure 4D

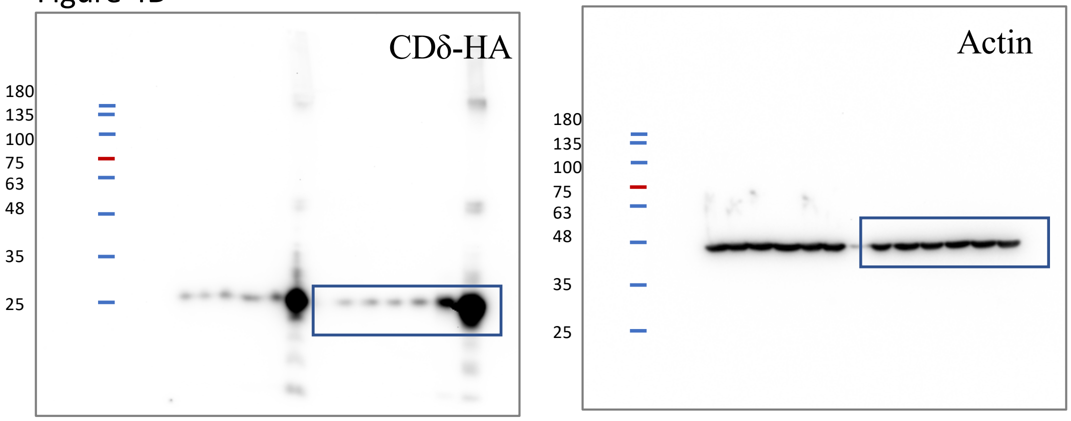

Figure 4C

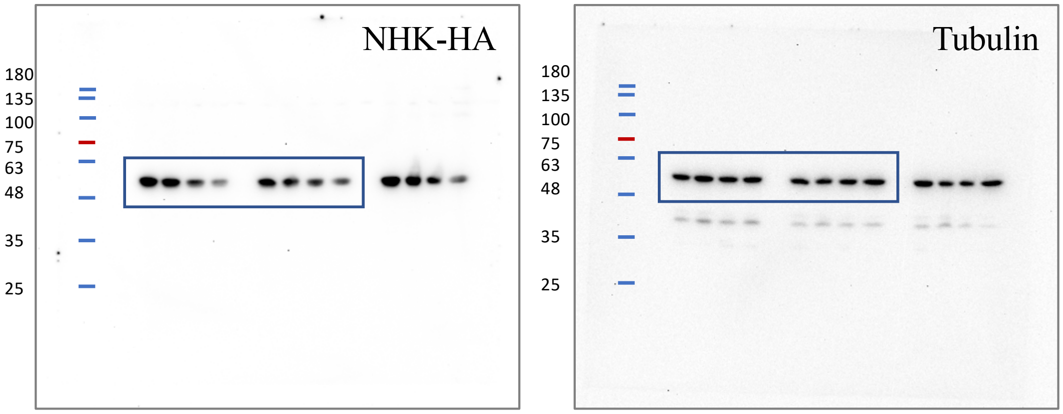

Figure 4E

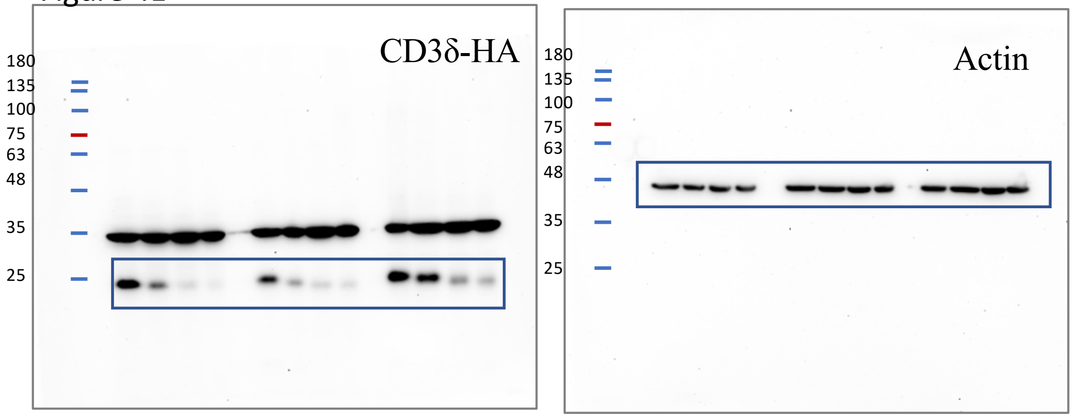

Figure 5

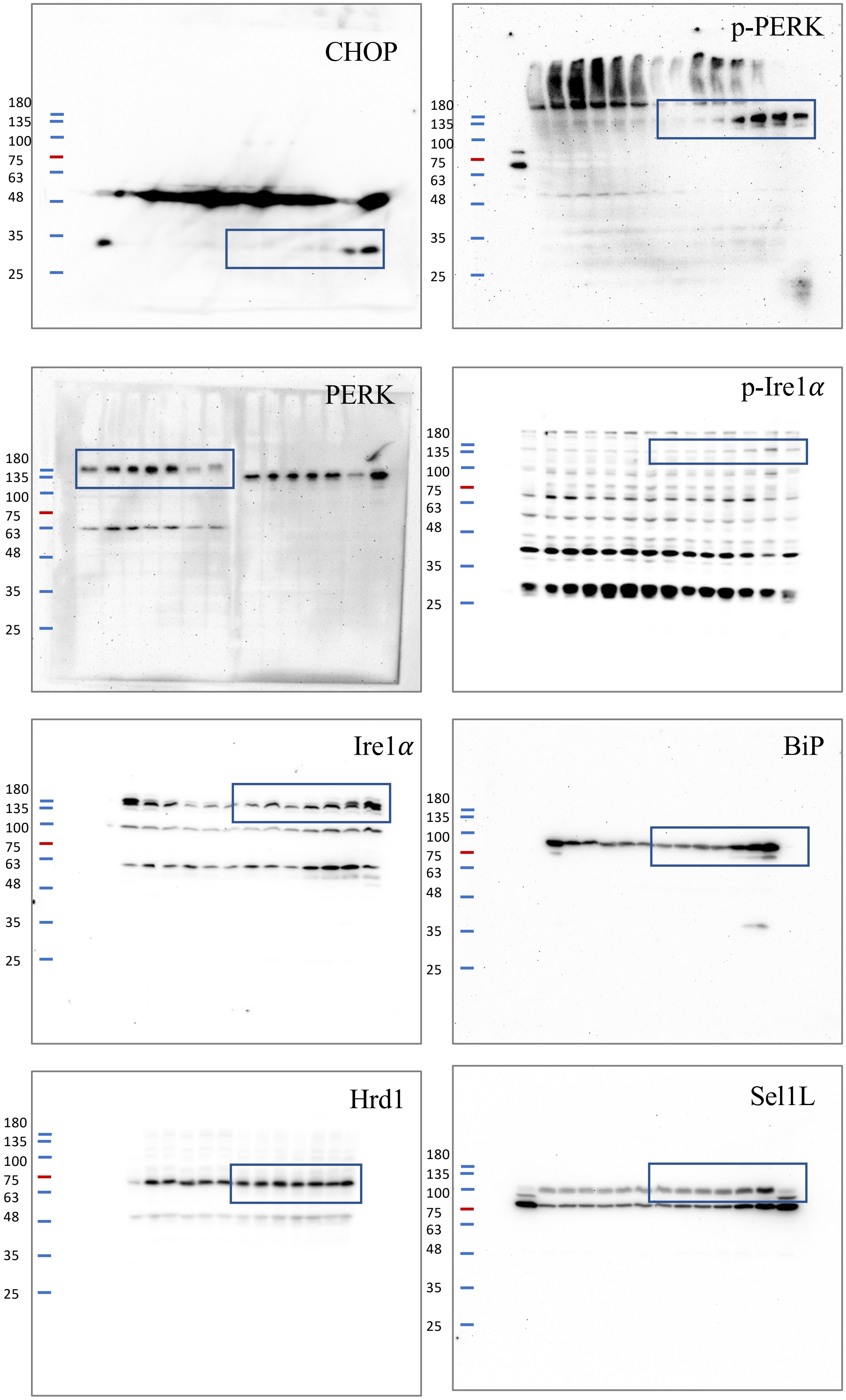
CHOP
180
135
100
75
63
48
35
25
p-PERK
180
135
100
75
63
48
35
25
PERK
180
135
100
75
63
48
35
25
p-Ire1α
180
135
100
75
63
48
35
25
Ire1α
180
135
100
75
63
48
35
25
BiP
180
135
100
75
63
48
35
25
Hrd1
180
135
100
75
63
48
35
25
Sel1L
180
135
100
75
63
48
35
25

Figure 5

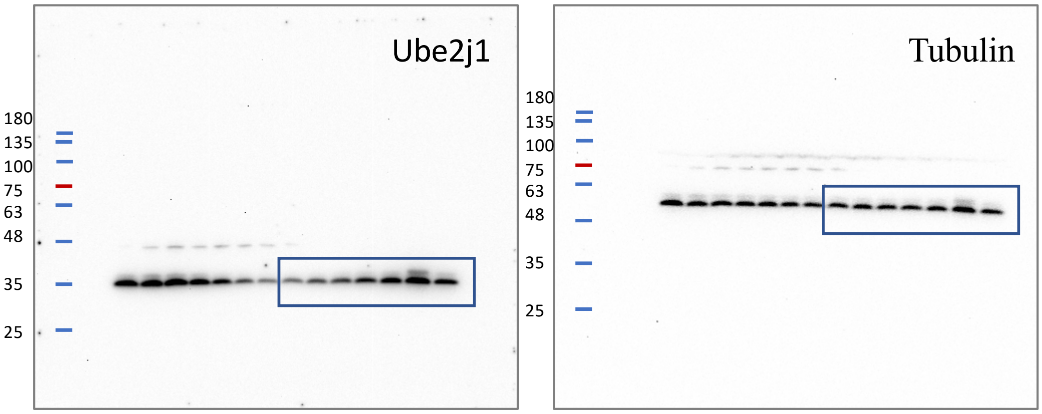
Ube2j1
180
135
100
75
63
48
35
25
Tubulin
180
135
100
75
63
48
35
25
